# Custom three-dimensional printed splint for postoperative rehabilitation of a terrible triad elbow injury

**DOI:** 10.1016/j.xrrt.2024.06.005

**Published:** 2024-06-25

**Authors:** Azim Huszar, Jason Thomas, Edris Adel, Charles Timon, Aidan O’Sullivan, Leonard O’Sullivan, John Tristan Cassidy

**Affiliations:** a University of Limerick, Limerick, Ireland; b University of Limerick Hospital Group, Limerick, Ireland; c Rapid Innovation Unit, School of Design and Confirm Smart Manufacturing Centre, University of Limerick, Limerick, Ireland; d Health Research Institute, University of Limerick, Limerick, Ireland

**Keywords:** 3D printing, Splint, Elbow, Terrible triad, Orthoses, Custom

The terrible triad elbow injury is characterized by a fracture of the radial head or neck, coronoid fracture, and elbow dislocation.^6^ These injuries are associated with significant elbow instability due to damage to the primary and secondary elbow stabilizers.^2^ Optimal outcomes are achieved via restoration of elbow stability and early, controlled postoperative rehabilitation. The rehabilitation protocol often involves early mobilization to prevent stiffness, as prolonged immobilization is a well-documented risk factor for postoperative elbow stiffness in these injuries.^10^ Typical splints used in the management of terrible triad injuries include those that maintain the elbow in flexion with or without pronation, providing necessary stability in the early postoperative course.^10^

## Methods

### Clinical presentation and surgical management

**Figure 1:**
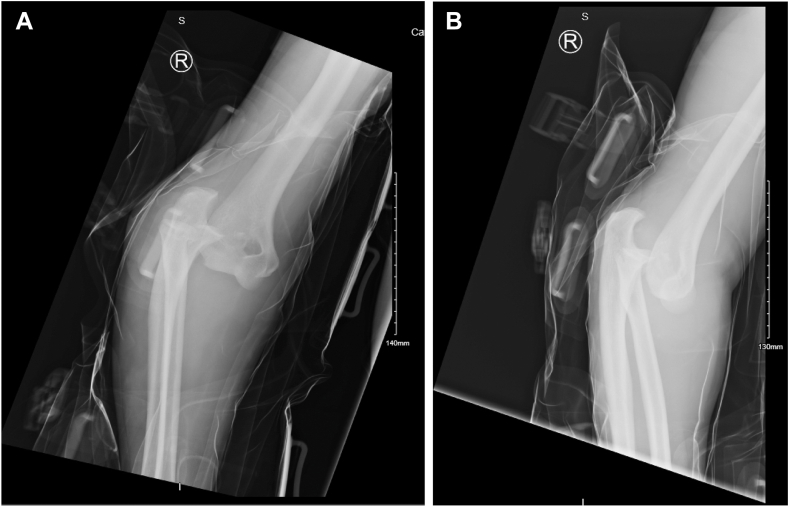
Initial views obtained in Emergency Department. (**A**) Anteroposterior view. (**B**) Lateral view.

**Figure 2:**
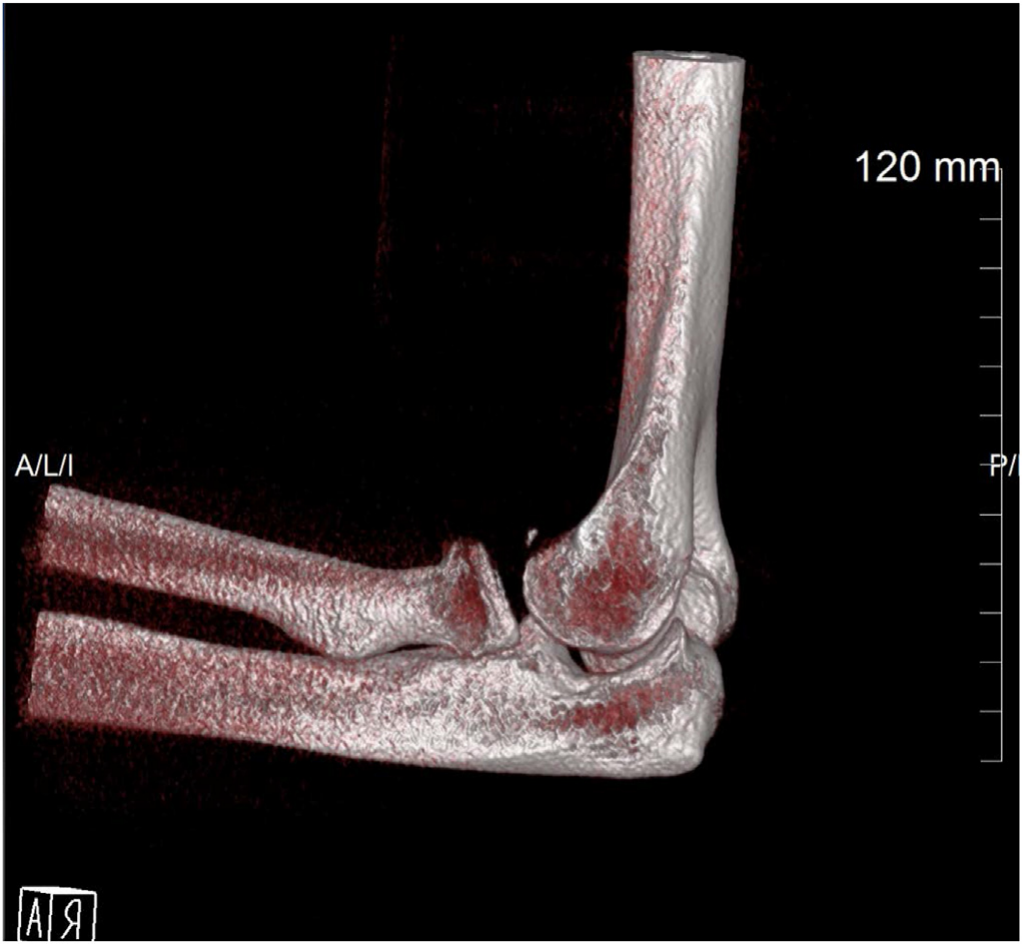
Preop 3D CT image. *3D*, three-dimensional; *CT*, computed tomography.

A 48-year-old left hand dominant male presented with a fracture-dislocation of the right elbow following a fall from two meters. There was no neurovascular compromise. The individual works as an electrician and plays recreational golf. Initial radiological evaluation demonstrated a terrible triad injury (Figs. 1 and 2) and closed reduction was performed in the emergency department. Further radiologic evaluation with computed tomography scanning revealed a Mason Type II radial head fracture^5^ and a Regan and Morrey I coronoid process fracture–primarily involving the lateral facet of the coronoid.^5^ The combination of the radial head fracture, lateral facet of the coronoid and combined lateral ulnar collateral injury (LUCL) and medial collateral injury–ligament injury confirmed by the degree of displacement on the x-rays resulted in the injury being classified as Wrightington Type C.^12^ The patient was referred for surgical repair, performed six days postinjury.

**Figure 3:**
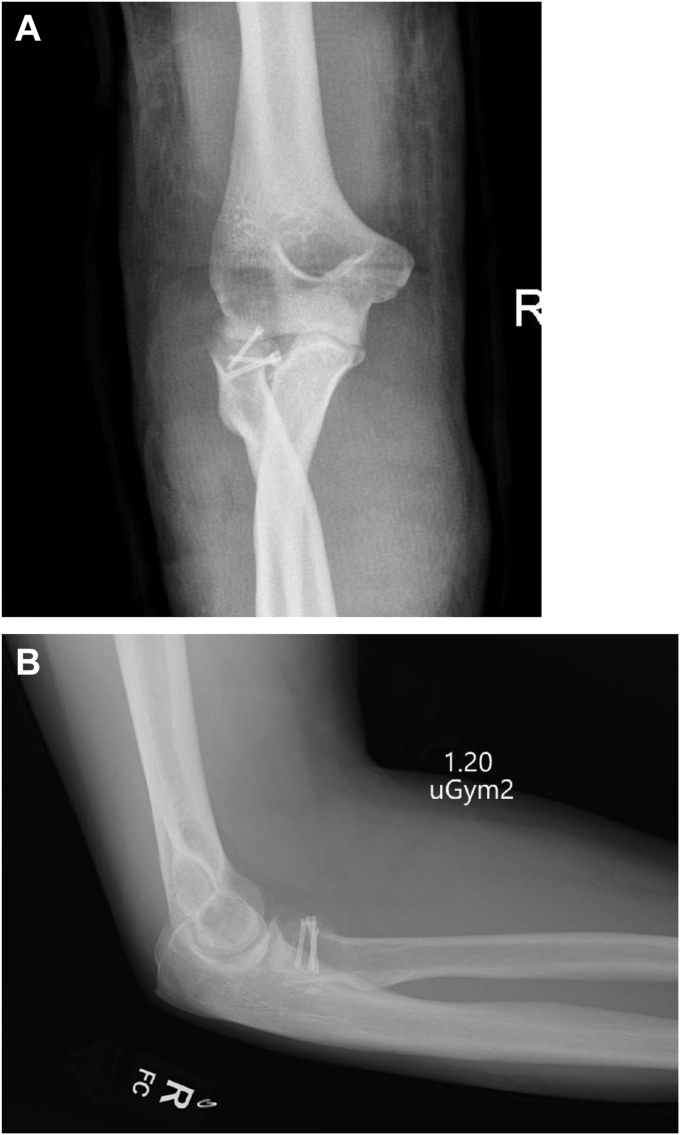
Postoperative Images postremoval of backslab and prior to application of the splint. (**A**) Anteroposterior view. (**B**) Lateral view.

Access was obtained using a lateral extensor digitorum communis split approach. The radial head fracture was fixed using 2 × 20 mm screws and a single 18 mm screw (Medartis, Basel, Switzerland) LUCL repair was achieved using suture anchors (2.9 mm all suture JuggerKnot Anchor; Zimmer Biomet, Warsaw, IN, USA) (Fig. 3). Due to the relative preservation of the coronoid height, fixation of the coronoid was considered unnecessary. Following LUCL repair, the elbow remained unstable, therefore repair of the MCL with suture anchors was performed using a medial incision (2.9 mm all suture JuggerKnot Anchor; Zimmer Biomet, Warsaw, IN, USA). In addition, transposition of the ulnar nerve was performed. Following surgical repair, the elbow was stable through extension of 30 degrees; however, both medial and lateral ligaments were needed to achieve on-table stability, postoperative splinting was considered mandatory. Immediate postoperative immobilization in flexion and full pronation was achieved using a backslab.

### Design and three-dimensional printing of elbow splint

Postoperative rehabilitation required a flexion-pronation splint. There was no suitable commercially available option for maintaining the forearm pronation while allowing for an adjustable range of motion (ROM). The Rapid Innovation Unit (RIU) from the University of Limerick was contacted and agreed to create a bespoke splint using three-dimensional (3D) printing technology.^7^^,^^8^

After consultation between RIU and the clinical team three fundamental design requirements were agreed which were to maintain the arm in a controlled position, to keep the patient’s wrist pronated, and to allow for a controlled ROM at the elbow.

The patients’ arm was held in the planned position of splinting (full pronation with 90 degrees of flexion) by the surgical team while a handheld 3D scanner was used to capture the geometry of the arm. The 3D model generated from the scan was used as a framework to build the bespoke elbow splint. By using the 3D scan data, an accurate match of the surface of patients’ arm is generated to avoid any excessive pressure points and create a comfortable fit which is customized to the patient.

The design for the brace was developed in Solidworks 3D modeling software (Waltham, MA, USA). The design consisted of two sections which supported the forearm, including a partial hand support, and the upper arm. These sections were connected via a hinged joint which limited the ROM to a specific degree with a mechanical end stop. This allowed for a controlled amount of extension at the elbow. The elbow brace was secured to the patients’ arm using commercially available compression wraps and supported with an over the neck sling.

A unique element of this design was the ability to change the end stop degree to customize the permitted ROM in tandem with the patients’ recovery. It was agreed that the initial ROM would be 10°, with the option to increase to a maximum of 45° over time.

**Figure 4:**
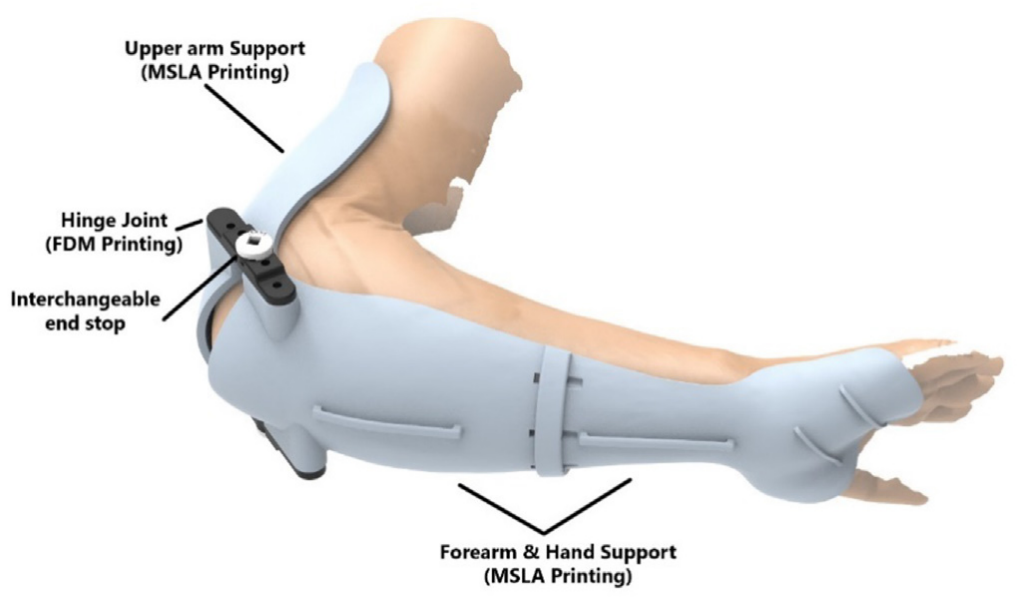
Computer Generated Rending of the 3D Printed Split.

An important consideration in the choice of 3D printing technology is the mechanical properties of the material; therefore, the completed design was 3D printed using a combination for fused deposition modeling and masked stereolithography apparatus printing (Fig. 3). The time from initial presentation of the patient to the RIU team to the delivery and fitting of the completed elbow brace was 7 days in total (Fig. 4). ROM exercises were immediately commenced postfitting of the brace. The total cost of materials for printing the splint was €17.98. The approximate combined cost of the 3D printers used was €1000 (€800 for the masked stereolithography apparatus printer and €200 for the fused deposition modelling printer).

### Outcome assessment and evaluation of splint

Patient outcomes included clinical ROM as measured by an upper limb specialist physiotherapist using a goniometer, the Disability of the Arm, Shoulder, and Hand score,^1^ the American Shoulder and Elbow Surgeons score,^1^ Subjective Elbow Value^9^ and the Quebec User Evaluation of Satisfaction with Assistive Technology (QUEST) score.^3^ The QUEST 2.0 score is a validated score to assess patient satisfaction with assistive technologies (orthotics) and associated services.^3^ The QUEST score is divided into two sections of eight questions regarding the assistive device and four questions regarding associated services (service delivery/repairs and servicing/professional services and follow-up services). The eight questions regarding the assistive device query the devices weight/ease of use/durability/safety and security/dimensions/comfort/ease of adjustment and effectiveness. Each criterium is scored from 0-5, with five being very satisfied and zero being not-at-all satisfied. Available scores for the assistive device section = 40 and services = 20, meaning the total max QUEST score is 60.

## Results

The 3D printed splint was fitted at day nine postop (Fig. 4). The patient and team were then able to rehabilitate the elbow as per standard protocol, with incremental increases to the range of extension by use of the hinges to limited extension at set angles. The custom-made splint was worn 24 hours per day, 7 days per week from week 2 to week 8 postoperatively. The custom-made splint was removed for regular performance of patient specific home exercise program. The custom-made splint held the patient in pronation. Full flexion was allowed, and extension was restricted to −60 degrees on initial application of the custom-made splint. Extension ROM was progressively increased by 15 degrees per week from week 2 to week 6 postoperatively. Supination exercises were commenced at 2 weeks postoperatively with the elbow flexed to 90 degrees in supine. Extension exercises were performed in pronation until week 8 postoperatively. Strength training using isometric cocontraction of the primary elbow stabilizers commenced at 2 weeks postoperatively. Progressive concentric and eccentric strength training using resistance bands and dumbbells commenced at 12 weeks postoperatively. Light functional activities commenced at 4 weeks postoperatively while in the custom-made splint and progressed to medium functional use in activities of daily living at 8 weeks postoperatively when the splint was removed. Phased return to work and sporting activities were allowed as tolerated from 16 weeks postoperatively.

**Figure 5:**
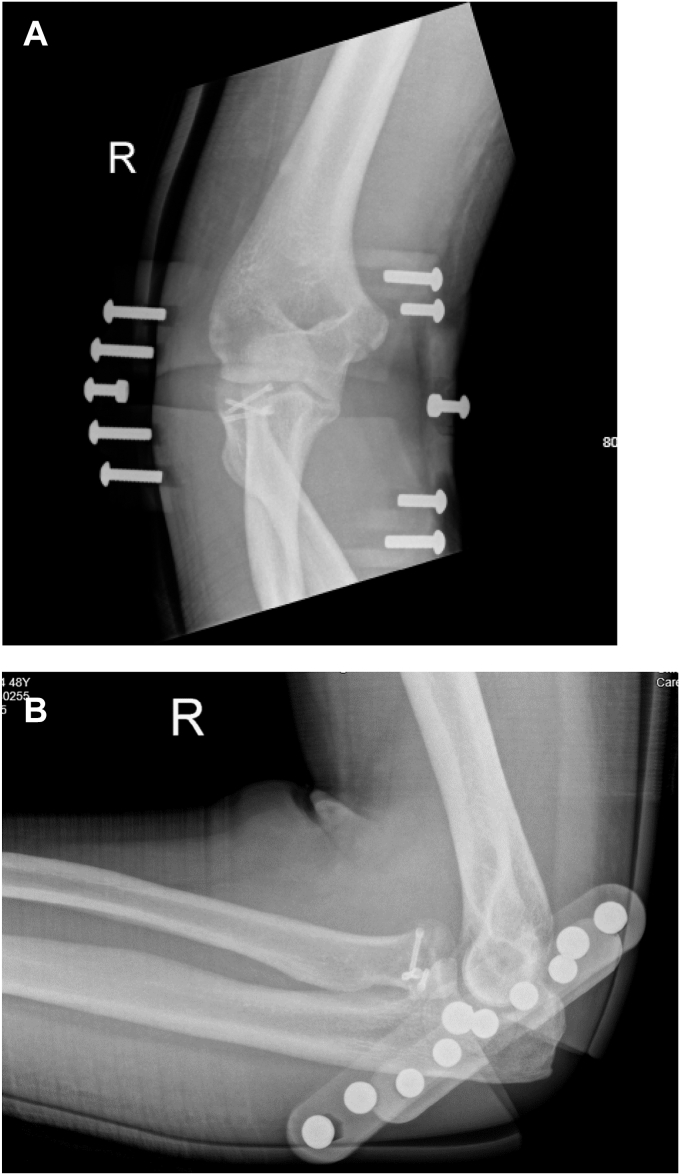
X-rays in splint. (**A**) Anteroposterior view. (**B**) Lateral view.

**Figure 6:**
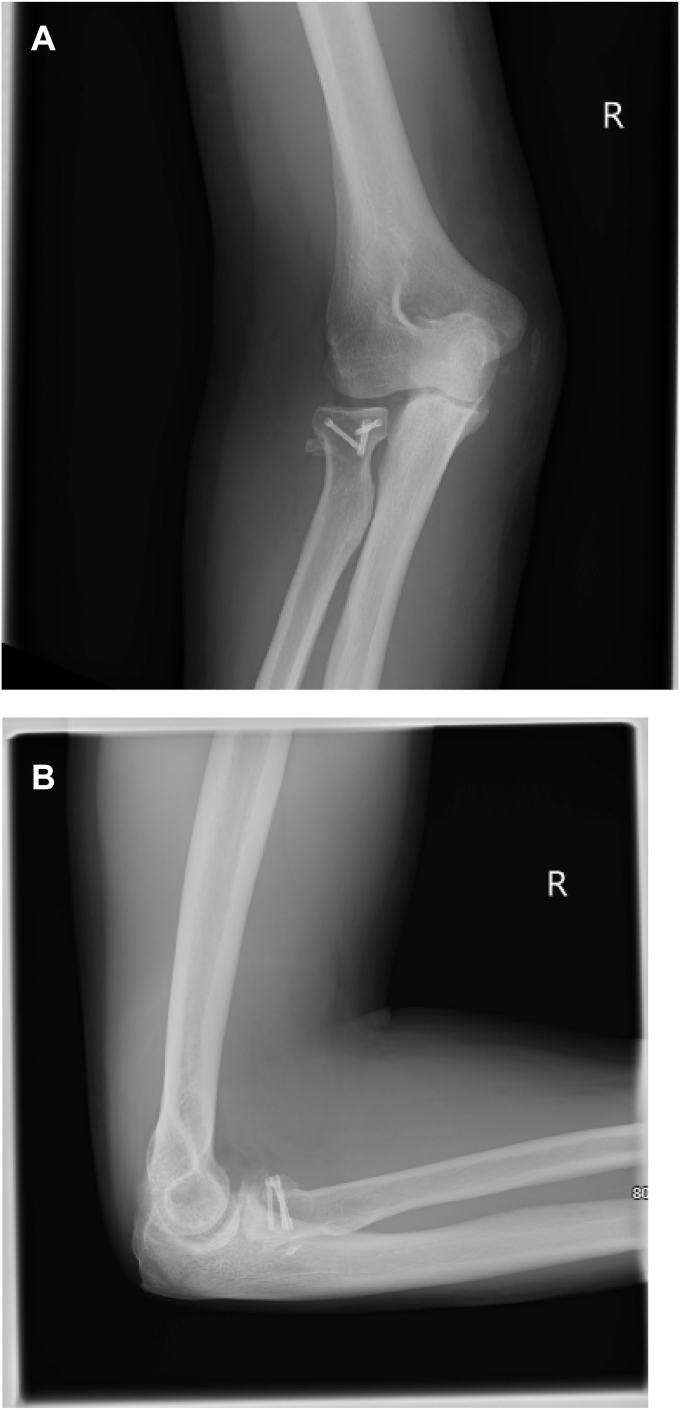
X-rays at one-year postop. (**A**) Anteroposterior view. (**B**) Lateral view.

At 6 months and one year the X-rays from clinic follow-up showed satisfactory healing alignment (Figs. 5 and 6). At final clinical assessment (one year postop) the ROM in degrees was: Extension −24, Flexion 138, Pronation 82, and supination 70. The Disability of the Arm, Shoulder, and Hand score was 14.16, the American Shoulder and Elbow Surgeons was 92 and the patient reported Subjective Elbow Value was 80/100. The QUEST 2.0 score was (36/40 for assistive device) + (20/20 for services) = 56/60. The two areas where the splint failed to reach full marks were safety and security (2/5) and durability (4/5). This was because the initial hinge failed and required repair.

## Discussion and conclusion

The availability of customized thermoplastic splint production in tandem with rehabilitation by subspecialist allied health professionals is not universal.^11^ In this case, a 3D custom elbow brace facilitated controlled rehabilitation in a case where postoperative stability was a concern, as illustrated by the need to repair the medial ligament in addition to the lateral ligament. Scanning in the clinical environment facilitated integration of the 3D mapping process into the patient’s clinical journey without the need for additional appointments. It also meant the splint could be measured and printed as soon as the wounds were mature enough to be braced and mobilized.

The patient reported high levels of satisfaction with the brace. Both clinical outcomes and patient reported outcomes were excellent and were comparable to results achieved in the literature.^4^ The splint performed well in most areas evaluated by the QUEST 2.0 score; however, the initial hinges produced by the 3D printing process were not robust enough and needed reenforcing. Consequently, the splint scored poorly in the “durability” section of the QUEST 2.0 score. The feedback was also expressed directly by the patient to the team.

Significant advantages of the 3D printing method of postoperative splinting include its low cost, speed of production and its role potential alternative option when specialist clinicians to produce a custom splint. To our knowledge, this is the first reported case of a personalized 3D printed splint in the setting of a terrible triad of the elbow. We propose it may be considered in the setting of complex elbow trauma where custom thermoplastic splinting is unavailable.

## Disclaimers:

Funding: This publication has emanated from research supported by 10.13039/501100001602Science Foundation Ireland (SFI) under Grant Numbers SFI 16/RC/3918 cofunded by the European Regional Development Fund.

Conflicts of interest: The authors, their immediate families, and any research foundation with which they are affiliated have not received any financial payments or other benefits from any commercial entity related to the subject of this article.

Patient consent: Obtained.
